# Accurate and Sensitive UHPLC–Tandem Mass Spectrometry Sequential Methods for Therapeutic Drug Monitoring of Aztreonam/Avibactam in Human Plasma

**DOI:** 10.3390/pharmaceutics18030377

**Published:** 2026-03-19

**Authors:** Ilaria Trozzi, Beatrice Giorgi, Riccardo De Paola, Milo Gatti, Federico Pea

**Affiliations:** 1 Clinical Pharmacology Unit, Department for Integrated Infectious Risk Management, IRCCS Azienda Ospedaliero-Universitaria di Bologna, 40138 Bologna, Italy; ilaria.trozzi@aosp.bo.it (I.T.); beatrice.giorgi@aosp.bo.it (B.G.); riccardo.depaola@studio.unibo.it (R.D.P.); milo.gatti2@unibo.it (M.G.); 2 Specialization School of Clinical Pharmacology and Toxicology, Alma Mater Studiorum Università di Bologna, 40138 Bologna, Italy; 3 Department of Medical and Surgical Sciences, Alma Mater Studiorum University of Bologna, 40138 Bologna, Italy

**Keywords:** aztreonam/avibactam, UHPLC–qTOF MS/MS, therapeutic drug monitoring, bioanalytical method validation, human plasma, high-resolution mass spectrometry, β-lactam/β-lactamase inhibitor antibiotics

## Abstract

**Background/Objectives**: The aztreonam/avibactam combination represents a promising therapeutic option for severe infections caused by multidrug-resistant Gram-negative pathogens, particularly in critically ill patients. Due to marked pharmacokinetic variability and the need to achieve joint pharmacokinetic/pharmacodynamic (PK/PD) targets of both agents, therapeutic drug monitoring (TDM) may play a pivotal role in optimizing treatment. This study aimed to develop and validate two rapid, accurate, and sensitive UHPLC–qTOF MS/MS sequential methods for quantifying aztreonam and avibactam in human plasma, suitable for routine clinical TDM. **Methods**: Plasma concentrations were determined by means of ultra-high-performance liquid chromatography coupled with quadrupole time-of-flight tandem mass spectrometry (UHPLC–qTOF MS/MS), operating in positive and negative electrospray ionization modes for aztreonam and avibactam, respectively. Sample preparation consisted of protein precipitation with isotopically labeled internal standards. The method’s validation was performed according to the European Medicines Agency guidelines, by assessing selectivity, linearity, precision, accuracy, recovery, matrix effects, carry-over, and stability. Clinical applicability was evaluated by reprocessing plasma samples, which were already previously collected for routine clinical practice from 20 hospitalized patients undergoing treatment with ceftazidime-avibactam plus aztreonam. **Results**: The methods showed excellent linearity (R^2^ ≥ 0.999) over ranges of 0.2–100 µg/mL for aztreonam and 0.1–50 µg/mL for avibactam. Lower limits of quantification were 0.2 µg/mL and 0.1 µg/mL, respectively. Intra- and inter-day precision and accuracy met the EMA criteria at all of the quality control levels. Extraction recovery exceeded 90% for both analytes, and matrix effects were effectively compensated by internal standards. Stability testing highlighted the need for careful sample handling, particularly for aztreonam under repeated freeze–thaw conditions. Clinical application revealed substantial inter-individual variability in steady-state concentrations. **Conclusions**: The validated UHPLC–qTOF MS/MS assays provide robust and sensitive sequential quantification of aztreonam and avibactam in human plasma, supporting TDM-guided dose optimization in clinical practice.

## 1. Introduction

Infections caused by carbapenem-resistant *Enterobacterales* (CPE) are an ever-growing major concern in several settings of critically ill patients [1,2]. Among CPE, those producing metallo-beta lactamases (MBLs) are particularly worrisome since the number of therapeutically effective weapons against MBL-related infections is quite limited [3,4]. In the recent past, international guidance and guidelines recommended combining aztreonam with ceftazidime-avibactam as one of the two first choices, together with cefiderocol, for treating MBL-producing *Enterobacterales* infections [5,6]. Specifically, aztreonam is inherently stable by itself to the MBLs, but unfortunately, it is susceptible to hydrolysis by serine β-lactamases, which are frequently co-produced by MBL-producing Gram-negative bacilli. To overcome this limitation, initially, aztreonam was extemporaneously combined with ceftazidime/avibactam to protect it from serine β-lactamase-mediated degradation, thereby restoring activity against MBL and serine β-lactamase co-producing *Enterobacterales* [7,8]. Recently, a new 3:1 fixed beta lactam/beta lactamase inhibitor combination (BL/BLIc) of aztreonam/avibactam was approved for this purpose and is currently being used in settings with high prevalence of these types of severe infections, occurring mainly in critically ill patients [9].

Aztreonam exhibits linear pharmacokinetics, moderate plasma protein binding, and is predominantly eliminated unchanged by the kidneys. Likewise, avibactam displays linear pharmacokinetics, low protein binding, and predominant renal elimination. Importantly, attaining a joint pharmacokinetic/pharmacodynamic (PK/PD) target is fundamental for optimal treatment with the aztreonam/avibactam combination. It has been shown that efficacy is granted whenever free aztreonam concentrations persisting for 60% of the dosing interval above the minimum inhibitory concentration (60%fT > MIC) are coupled with free avibactam concentrations persisting for 50% of the dosing interval above a target concentration of 2.5 mg/L (50%fC_T_ > 2.5 mg/L) [10,11,12,13,14]. Unfortunately, like with other types of BL/BLIc, aztreonam/avibactam is also prone to high inter- and intra-individual pharmacokinetic variability in the critically ill patients. Factors such as sepsis-associated tissue capillary-leakage and/or transient acute kidney injury and/or augmented renal clearance may affect both the volume of distribution and the renal clearance of either aztreonam or avibactam [13,15]. This may be especially relevant in affecting an optimal joint PK/PD target attainment because, being the renal clearance of avibactam much faster than that of aztreonam, the 3:1 proportion of the aztreonam/avibactam concentrations present in the vial may vary in the critically ill patients as a consequence of the aforementioned conditions [16]. Consequently, this may lead to subtherapeutic or potentially toxic plasma concentrations, thus compromising efficacy or safety when standard dosing regimens are used. In this context, therapeutic drug monitoring (TDM) may be a valuable clinical tool for personalizing antibiotic therapy with the intent of improving the likelihood of optimal joint PK/PD target attainment and consequently the clinical outcome [17,18,19]. In this regard, implementing robust and reliable analytical methods capable of accurately quantifying both aztreonam and avibactam in biological matrices is an absolute pre-requisite for properly dealing with real-time TDM-guided dosing adaptation of aztreonam/avibactam in critically ill patients. Liquid chromatography coupled with tandem mass spectrometry (LC-MS/MS) is currently the gold standard approach for measuring antibiotics in plasma or serum, owing to its high sensitivity, specificity, and analytical robustness [20,21,22,23]. Some LC-MS/MS and UHPLC-MS/MS methods have been reported for the simultaneous determination of multiple β-lactam antibiotics, including aztreonam and avibactam, in human plasma, and have been successfully applied in the context of clinical TDM [24,25]. These methods generally comply with international bioanalytical validation guidelines and may allow for rapid and reliable quantification in routine laboratory practice. However, methods for quantifying aztreonam/avibactam in human plasma by means of high-resolution mass spectrometry platforms, namely UHPLC–quadrupole time-of-flight (qTOF) MS/MS, are still lacking. HRMS-based approaches may have several advantages compared to the traditional mass spectrometry techniques, namely high mass accuracy, enhanced selectivity and sensitivity, and the possibility of retrospectively enquiring the system about data just previously acquired, all of which may be especially valuable in complex clinical matrices and in the context of multi-analyte or exploratory analyses [26]. The aim of this study was to describe the development and validation of two rapid UHPLC–qTOF MS/MS sequential methods for the accurate and precise quantification of aztreonam/avibactam in human plasma. The proposed methods are intended to support real-time TDM in clinical practice, providing a reliable analytical tool for dose individualization and optimization of aztreonam/avibactam therapy.

## 2. Materials and Methods

### 2.1. Chemicals and Reagents

**Figure 1:**
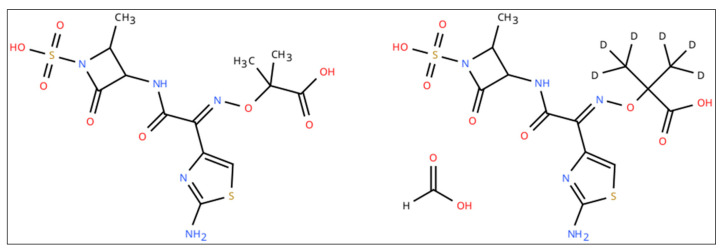
Chemical structure of aztreonam (**left**) and [2H6]-aztreonam formate salt (**right**).

**Figure 2:**
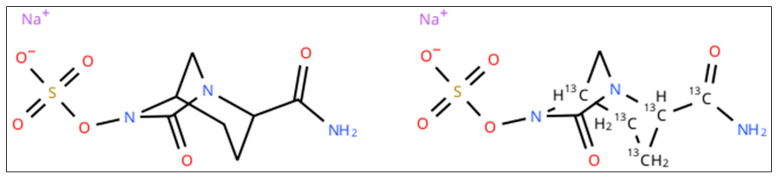
Chemical structure of avibactam (**left**) and [13C5]-avibactam sodium salt (**right**).

Lyophilized standard of aztreonam, [13C5]-avibactam sodium salt and [2H6]-aztreonam formate salt (chemical structures shown in Figure 1 and Figure 2) were purchased from Alsachim Shimadzu Chemistry & Diagnostics (Illkirch, France). Avibactam powder (Figure 1) and drug-free human plasma were provided by Merck KgaA (Darmstadt, Germany). Liquid chromatography–MS/MS-grade reagents were purchased from Thermo-Fisher Scientific (Milan, Italy).

### 2.2. Stock Solutions, Calibrators and Quality Control Samples

Aztreonam and avibactam stock solutions at a concentration of 1 mg/mL and 5 mg/mL, respectively, were prepared by dissolving the analyte powders in DMSO. Solutions of calibrators for the calibration curves and of independent quality controls (QCs) were prepared in MilliQ water (Sigma-Aldrich, Merck, Darmstadt, Germany) at different concentrations as serial dilutions from the respective stock solution, by spiking drug-free plasma in each of them. Calibration ranges covered from 0.2 to 100 μg/mL (calibration points: 0.2–1–5–10–25–50–100 μg/mL) for aztreonam and from 0.1 to 50 μg/mL (calibration points: 0.1–1–2.5–5–10–25–50 μg/mL) for avibactam. QC samples were prepared at the concentrations of 2.5 μg/mL (LQC), 20 μg/mL (MQC) and 80 μg/mL (HQC) for aztreonam and 1.5 μg/mL (LQC), 8 μg/mL (MQC) and 40 μg/mL (HQC) for avibactam. The dynamic ranges of the calibration curves were based on the expected therapeutic plasma concentrations of the analytes in critically ill patients receiving treatment by extended infusion over 3 h. A mixed solution containing 1 μg/mL of [2H6]-aztreonam formate salt plus 1 μg/mL of [13C5]-avibactam in methanol was used as internal standard (IS). All solvents and matrix solutions were stored at −80 °C.

### 2.3. Sample Pre-Treatment

For sample preparation, 10 µL of human plasma diluted with 80 µL of ultrapure water was added to 300 µL of the methanol internal standard (IS) solution. The mixture was kept at room temperature, vortexed for 15 s and then centrifuged at 13,000 rpm for 5 min. After centrifugation, 100 µL of the clear supernatant was transferred to an autosampler vial, and a 2 µL aliquot was injected into the LC–MS/MS system.

### 2.4. Instrumentation and Analysis

Samples were analyzed by means of a Shimadzu Nexera U-HPLC system equipped with binary pumps for mobile-phase delivery and coupled with a Quadrupole Time-of-Flight mass spectrometer X500 QTOF (AB SCIEX, Marlborough, MA, USA). Data were acquired in Multiple Reaction Monitoring (MRM) mode, with electrospray ionization (ESI) operating in the positive mode for the analysis of aztreonam and in the negative mode for that of avibactam. Separation was performed by means of an Agilent Poroshell 120 EC-C18 (2.1 × 50 mm, 1.9 µm) column for aztreonam and a Poroshell 120 PFP (2.1 × 50 mm, 2.7 µm) column for avibactam, ensuring the elution of the analytes at a specific and reproducible retention time. Both columns were kept at 45 °C and coupled with an autosampler kept at 4 °C. Analyte separation was achieved by using a binary pump system with a linear gradient elution from mobile-phase A [water with 0.2% formic acid, *v*/*v*] to mobile-phase B [methanol/acetonitrile 50:50 with 0.2% formic acid, *v*/*v*] at a flow rate of 0.5 mL/min (Table 1 and Table 2).

Data acquisition was carried out in MRM mode, with electrospray ionization (ESI) operating in positive or in negative mode, as needed. The MS/MS parameters for positive polarization and for negative polarization are summarized in Table 3 and Table 4, respectively. Chromatographic data acquisition, peak integration, and quantification were performed by means of the Sciex OS 3.1.6 software (AB SCIEX, Marlborough, MA, USA).

### 2.5. Methods Validation

The two methods were validated in accordance with the European Medicines Agency (EMA) guidelines for bioanalytical method validation [27]. Validation parameters included selectivity, linearity, accuracy, precision, lower limit of quantification (LLOQ), recovery, matrix effects and stability.

#### 2.5.1. Selectivity and Carry-Over

Twenty plasma samples were tested to check for the absence of both the analytes at the retention times and the deuterated IS of any response attributable to endogenous or exogenous components of the matrix. The carry-over was assessed by injecting a blank sample immediately after finishing the highest calibrator run (ULoQ), and it was considered negligible whenever a signal intensity < 20% of that of the LLOQ was detected, as recommended by the EMA guidelines [27].

#### 2.5.2. Linearity and Limit of Quantification (LoQ)

Calibration curves were constructed by means of weighted (1/x) linear regression. Linearity assessment was based on back-calculated concentrations of the calibration points deviating maximum ±15% from the nominal values (±20% at the LLOQ), in agreement with the ICH M10 guidelines. Acceptance of calibration curve linearity was verified with correlation coefficients (R^2^) ≥ 0.999. The limit of quantification (LoQ) was defined as the lowest calibrator within the dynamic range (namely 0.2 µg/mL for aztreonam and 0.1 µg/mL for avibactam) exhibiting a signal-to-noise ratio (S/N) > 10.

#### 2.5.3. Precision and Accuracy

Precision (expressed as mean CV%) and accuracy (expressed as mean BIAS%) were established by analyzing the LLOQ, the LQC, the MQC, and the HQC five times within the same day (intra-day) across three separate inter-day analytical sessions.

#### 2.5.4. Matrix Effect and Extraction Recovery

The matrix effect (ME) and the extraction recovery (ER) were assessed at the three quality control (QC) levels (namely, LQC, MQC, and HQC). The ME was assessed quantitatively by comparing plasma samples spiked with aztreonam and avibactam to water samples spiked with the same analytes. The analyte ratios obtained from plasma and water were compared, and the mean values and coefficients of variation (CVs) were calculated across six independent batches. The ER was determined by comparing plasma extracts spiked with both the analytes and the IS with those spiked with only the IS and expressed as a percentage. The compensated ME was calculated as the analyte-to-IS area ratio, and to be considered negligible, it had to be <15%.

#### 2.5.5. Stability

The stability of aztreonam and of avibactam in human plasma was assessed at three concentration levels within the calibration range (LQC, MQC, and HQC) under different storage conditions. In accordance with laboratory needs and routine practice, the tests assessed the stability of the extracts stored at 4 °C for 24 h and for 5 days, as well as that of the matrix samples after three complete freeze–thaw cycles from −80 °C to 25 °C. Measured sample concentrations before and after storage were compared with nominal values, and the stability was defined as acceptable if the effective concentrations were within ±15% of the nominal concentrations.

#### 2.5.6. Application to Clinical Samples

The developed LC–MS/MS sequential methods were validated by measuring the plasma concentrations of aztreonam/avibactam in reprocessed samples which were already previously collected for routine clinical practice from 20 hospitalized patients undergoing treatment with ceftazidime-avibactam plus aztreonam according to the retrospective observational study approved by the Ethics Committee of the IRCCS Azienda Ospedaliero-Universitaria di Bologna (No. 442/2021/Oss/AOUBo on 22 June 2021, EM232–2022/Oss/AOUBo on 16 March 2022, and EM449–2023/Oss/AOUBo on 17 April 2024). Informed written consent was waived due to the retrospective and observational nature of the study, according to the article 110-bis comma 4 of Legislative Decree No. 196/2003 rev. 2024 of Italian Law regulating retrospective study.

## 3. Results

### 3.1. Optimization of Analytical Conditions

Single-charge positive and negative ion mass transitions were selected for optimal sensitivity and specificity by scrutinizing the MS/MS fragmentation pattern spectra of the analytes and by comparing them with those reported in the literature [21,23,28]. The specific MRM transition parameters are summarized in Table 5.

**Figure 3:**
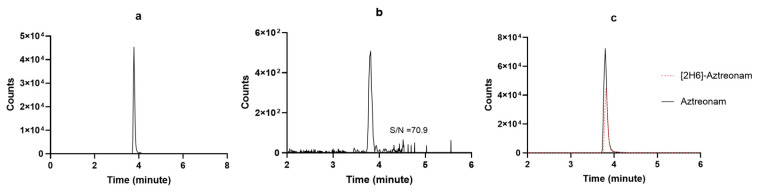
MRM chromatograms of (**a**) [2H6]-aztreonam formate salt; (**b**) a LLOQ aztreonam sample with the respective S/N ratio; (**c**) a real patient sample (black continuous line) overlayed with the IS peak (red dotted line).

**Figure 4:**
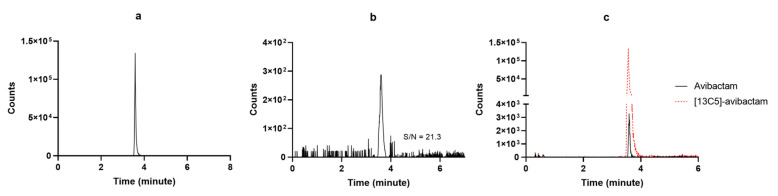
MRM chromatograms of (**a**) [13C5]-avibactam sodium salt; (**b**) a LLOQ avibactam sample with the respective S/N ratio; (**c**) a real patient sample (black continuous line) overlayed with the IS peak (red dotted line).

Method development started by testing preliminarily if the chromatographic conditions available in our lab for measuring other BL/BLIc could have fit for our purpose. Since this was not the case, we applied several adaptations and the final chromatographic conditions were definitely selected once reaching high-resolution MS detection based on adequate retention times, peak symmetry, signal intensity, and reproducibility under gradient conditions. The chosen stationary phases provided the best overall balance between chromatographic performance and analytical robustness for each analyte within the selected instrumental configuration. The selected LC elution conditions were good enough for granting high quality and sharp shape of the chromatographic peaks, as shown in Figure 3 and Figure 4. The retention times were constant and reproducible, thus supporting an optimal inter-run column reconditioning. The MRM chromatograms obtained by analyzing a drug-free plasma sample (blank), detecting only the specific signal of [2H6]-aztreonam formate salt and of [13C5]-avibactam, are depicted in Figure 3a and Figure 4a, respectively. This confirms both the high specificity of the MRM transitions and the purity of the IS solutions. The MRM chromatograms obtained by analyzing the LLOQ samples of aztreonam and avibactam are depicted in Figure 3b and Figure 4b, respectively. The S/N ratios were high (70.9 and 21.3, respectively), thus confirming the high sensitivity of the methods. Real sample MRM chromatograms of aztreonam and avibactam (Figure 3c and Figure 4c) show that for both peaks, shapes were sharp and the resolutions were optimal, with no isobaric interference coming from interferences generated by endogenous components and/or by any other drug. Noteworthy, the IS peak areas almost overlapped with those of the analytes concentrations expected in patients.

### 3.2. Method Validation

**Figure 5:**
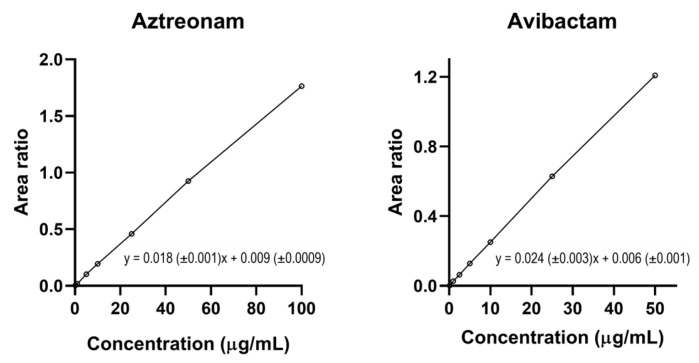
Calibration curves of aztreonam and of avibactam and their respective linear equation based on 7 incremental calibrators each obtained by plotting the aztreonam/[2H6]-aztreonam and the avibactam/[13C5]-avibactam area ratio over concentration, respectively.

The sequential methods were validated according to the EMA guidelines showed excellent selectivity and specificity with no interfering peaks at the optimized chromatographic conditions. Thanks to the small injection volume (2 µL), the carry-over was absent. The linear regression fit of the calibration curves was excellent (with R^2^ ≥ 0.999) for both aztreonam and avibactam within the respective optimized dynamic range (0.2–100 μg/mL for aztreonam and 0.1–50 μg/mL for avibactam), as shown in Figure 5. The results of the intra-day and inter-day test of precision and accuracy were always within the acceptable ±15% ranges for both aztreonam and avibactam, as recommended by the EMA Guidelines [27] (Table 6 and Table 7, respectively).

The findings of %ME, %IS-normalized ME, and %ER of the LQC, the MQC, and the HQC of aztreonam and avibactam are summarized in Table 8 and Table 9, respectively. At all of the tested QC levels, the %ME was negative for aztreonam with a moderate signal suppression, whereas it was positive for avibactam with a slight signal increase. After IS normalization, the %ME values matched the criteria established by the EMA for validation. The %ER was very high for both aztreonam and avibactam, exceeding 90% at all of the tested concentrations. These findings may support the need for an IS to provide an accurate quantification across the entire tested dynamic range.

The findings concerning the test of stability of aztreonam and avibactam are summarized in Table 10 and Table 11, respectively. Specifically, when the LQC, the MQC and the HQC were kept in the autosampler at 4 °C, aztreonam was stable for up to three days and avibactam for up to five days. Regarding the freeze–thaw stability, aztreonam was completely stable after the first cycle, showed borderline stability after the second cycle, and was markedly unstable after the third cycle. Conversely, avibactam easily withstood up to three freeze–thaw cycles.

### 3.3. Clinical Application

**Figure 6:**
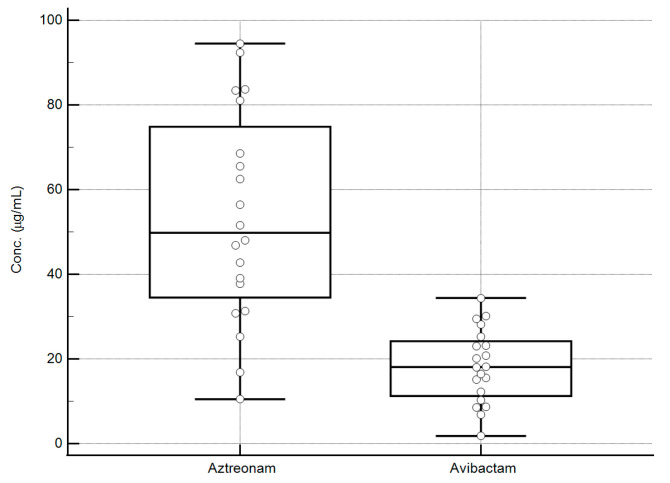
Box and whisker plots of the steady-state concentration of aztreonam/avibactam measured in the plasma of 20 hospitalized patients. The values obtained range from 10.5 to 94.5 µg/mL for aztreonam and from 1.8 to 34.4 µg/mL for avibactam, falling well within the validation ranges of the analytical methods. Box represents median and interquartile range for each analyte, external lines represent the range of concentrations (min–max) for each analyte, whereas the circles represent the different concentrations measured for each analyte.

All aztreonam/avibactam concentrations measured in the critically ill patients’ plasma using the developed LC-MS/MS sequential methods were within the validated dynamic ranges. The distribution of the plasma concentrations of the analytes was quite widespread, as shown in the box and whisker plots of Figure 6. This may support the usefulness of these methods for applying real-time TDM-guided optimization of aztreonam/avibactam therapy.

## 4. Discussion

In this study, two sensitive, accurate, and robust UHPLC–qTOF MS/MS sequential methods were developed and fully validated for quantifying aztreonam/avibactam in human plasma, with the specific aim of supporting TDM in clinical practice. The analytical performance achieved, particularly in terms of sensitivity, precision, and rapid turnaround time, compares favorably with previously published LC-MS/MS methods for β-lactam antibiotics, which are increasingly used to guide individualized dosing strategies in critically ill patients [17,18]. The linearity over a wide range of concentrations of the calibration curves and the specific LLOQ of these methods, namely among the lowest reported, may enable reliable measurement across wide concentration ranges encompassing both subtherapeutic and potentially supratherapeutic levels, regardless of using small plasma volumes [24,29,30,31]. The relevance of such analytical sensitivity is underscored by the pronounced intra- and inter-individual pharmacokinetic variability that aztreonam/avibactam—and likewise other BL/BLIc—may exhibit in hospitalized critically ill patients, driven by altered renal clearance and changes in volume of distribution [10,32,33]. Additionally, since the efficacy of aztreonam/avibactam is strictly dependent on the attainment of an optimal joint PK/PD target, the different variations in the renal clearance of avibactam compared to that of aztreonam potentially occurring in the critically ill patients could lead to major changes in the 3:1 aztreonam/avibactam proportion present in the vial, thus causing suboptimal joint PK/PD target attainment [12,13]. In this context, the present methods provide a valuable tool for real-time dose adaptation, complementing emerging clinical evidence supporting β-lactam TDM as a means to improve clinical outcomes and reduce the risk of therapeutic failure. It is noteworthy that the use of isotopically labeled internal standards enabled effective correction for plasma matrix variability, namely a major critical issue occurring in heterogeneous populations such as critically ill patients [29]. This may further improve the robustness and the reliability of the developed methods. Noteworthy, our methods of measuring aztreonam/avibactam were developed by using a high-resolution qTOF platform combined with a UPLC system. This approach may offer several advantages compared with the only other LC-MS/MS method available in the literature, which was based instead on a conventional triple-quadrupole approach [24]. Specifically, the high accuracy of mass detection may enhance the possibility of properly differentiating the target analytes from potential matrix interferents, even when the latter have very similar m/z values [26]. Furthermore, the quantification limits are significantly lower for both analytes, being even one order of magnitude lower for aztreonam, thus enabling the ability to work with small sample volumes. Finally, the qTOF platform, by recording a full scan of all of the ions generated from the sample and not only the signals of those of specific interest, may allow for the re-examination of the saved data files at a later stage. This could be especially valuable, for example, whenever searching for any other new potential interferent not originally taken into consideration. This may be especially valuable in the presence of complex clinical matrices and/or in the case of patients receiving multiple concomitant therapies, in which the risk of analytical interferences might be non-negligible [21]. The successful application of this method on clinical samples collected from patients having a wide inter-individual variability of concentrations may support the suitability and the utility of the methods for routine clinical use, especially considering the ever-growing role that aztreonam/avibactam has acquired in the treatment of MBL-producing *Enterobacterales*-related infections [25].

We recognize that the need to use two sequential chromatographic methods based on different stationary phases for maximizing analytical robustness and reliability could be a limitation. Unfortunately, the simultaneous measuring of aztreonam and avibactam on a single column was unfeasible due to the markedly different physicochemical properties and retention behaviors of the two analytes. However, it should not be overlooked that both the sample preparation protocol and the UHPLC–qTOF MS/MS analytical platform were shared by the two methods, and this may be time-saving when operating in routine therapeutic drug monitoring. Incurred sample reanalysis (ISR) of real-world clinical samples used for validating the analytical methods was not performed. We are aware that the ICH M10 guidelines recommend using this approach for testing bioanalytical methods supporting pharmacokinetic studies during pivotal clinical trials. Our aim was simply to validate the analytical method by showing that it was feasible for measuring residual clinical samples coming from routine therapeutic drug monitoring. Although we recognize that ISR could have added some value, its absence does not affect in any way the reliability, robustness and reproducibility of our sequential methods, as witnessed by the positive outcome of all the procedures for bioanalytical method validation recommended by the EMA guidelines.

Finally, it should not be overlooked that the incomplete stability of the two analytes under different testing conditions needs particular attention and may limit the application of these methods for real-time TDM in some settings. Both aztreonam and avibactam were stable for at least 3 days when kept at +4 °C. This means that after collecting blood samples from the patients, it is preferable to keep them at +4 °C during transport. At the same time, keeping sample extracts at +4 °C in the refrigerator could allow for reanalysis several times in the subsequent 2–3 days whenever needed. Conversely, the freeze–thaw tests showed that although avibactam withstood three freeze–thaw cycles, unfortunately, aztreonam was stable for only one thawing cycle. Consequently, whenever samples are delivered from a remote hospital to a hub lab, it is recommended that blood samples should be centrifuged locally and the supernatant, after being separated, should be stored in dry ice during transport until processing, so that they may undergo a maximum of one freeze–thaw cycle.

## 5. Conclusions

In conclusion, the two developed and validated UPLC–qTOF MS/MS sequential methods for measuring aztreonam/avibactam in human plasma were rapid, sensitive, and accurate, granting high performance, reliability, and short running times. Consequently, this may support their use as a robust bioanalytical platform suitable for real-time TDM-guided dose adjustment of aztreonam/avibactam in routine clinical practice.

## Figures and Tables

**Table 1:** UHPLC method for aztreonam by means of the 120 EC-C18 column.

Time (min.)	A (%)	B (%)	Flow (mL/min.)
0.00	98	2	0.5
0.20	98	2	0.5
4.30	40	60	0.5
4.40	2	98	0.5
5.70	2	98	0.5
5.80	98	2	0.5
8.00	98	2	0.5

**Table 2:** UHPLC method for avibactam by means of the 120 PFP column.

Time (min.)	A (%)	B (%)	Flow (mL/min.)
0.00	98	2	0.5
0.10	98	2	0.5
5.00	0	100	0.5
7.00	0	100	0.5
7.10	98	2	0.5
12.0	98	2	0.5

**Table 3:** MS/MS parameters for positive polarization.

**Ion source gas 1 pressure**	**Ion source gas 2 pressure**	**Curtain gas pressure**	**CAD gas**	**Gas** **temperature**	**Polarity**	**Spray** **voltage**
45 psi	55 psi	35 psi	7	550 °C	Positive	5500 V
**TOF start-stop mass**	**Accumulation time**	**Declustering potential**	**DP spread**	**Collision** **energy (CE)**	**CE spread**	
250–850 Da	0.25 s	40 V	0 V	5 V	0 V	

**Table 4:** MS/MS parameters for negative polarization.

**Ion source gas 1 pressure**	**Ion source gas 2 pressure**	**Curtain gas pressure**	**CAD gas**	**Gas** **temperature**	**Polarity**	**Spray** **voltage**
40 psi	45 psi	35 psi	7	450 °C	Negative	−4500 V
**TOF start-stop mass**	**Accumulation time**	**Declustering potential**	**DP spread**	**Collision** **energy (CE)**	**CE spread**	
100–400 Da	0.1 s	−40 V	0 V	−5 V	0 V	

**Table 5:** Specific multiple reaction monitoring (MRM) transition parameters used for aztreonam, avibactam, and their respective internal standards.

Analyte	Retention Time (min.)	Precursor Ion—Quantifier (*m*/*z*)	Precursor Ion—Qualifier (*m*/*z*)	Accumulation Time (ms)	DP (V)	CE (V)
Aztreonam	3.80	436.05940	313.06080	250	20	20
[2H6]-aztreonam formate salt	3.80	442.09660	319.09860	250	20	20
Avibactam	3.30	264.03080	95.95280	50	−40	−30
[13C5]-avibactam	3.30	269.04730	95.95260	50	−40	−30

**Table 6:** Intra-day and inter-day average (Avg) precision and accuracy evaluated at four concentration levels (LLOQ, LQC, MQC, and HQC) for aztreonam.

QC Levels	Nominal Conc. (µg/mL)	Intra-Day (*n* = 5)			Inter-Day (*n* = 3)		
		Avg. Conc. (µg/mL)	Avg. Precision (CV%)	Avg. Accuracy (Bias %)	Avg. Conc. (µg/mL)	Avg. Precision (CV%)	Avg. Accuracy (Bias %)
LLOQ	0.2	0.22	7.64	10.00	0.21	5.59	3.33
LQC	2.5	2.76	5.60	10.40	2.80	1.44	12.13
MQC	20	21.8	3.37	9.00	22.17	1.55	10.83
HQC	80	80.68	1.94	0.85	76.39	6.27	−4.52

**Table 7:** Intra-day and inter-day average (Avg) precision and accuracy evaluated at four concentration levels (LLOQ, LQC, MQC, and HQC) for avibactam.

QC Levels	Nominal Conc. (µg/mL)	Intra-Day (*n* = 5)			Inter-Day (*n* = 3)		
		Avg. Conc. (µg/mL)	Avg. Precision (CV%)	Avg. Accuracy (Bias %)	Avg. Conc. (µg/mL)	Avg. Precision (CV%)	Avg. Accuracy (Bias %)
LLOQ	0.1	0.12	9.93	13.71	0.11	10.12	−12.80
LQC	1.5	1.56	8.70	9.56	1.52	7.98	11.23
MQC	8	8.3	5.71	11.40	8.17	4.54	12.56
HQC	40	40.68	3.85	2.36	40.39	9.46	6.18

**Table 8:** Average matrix effect (ME%), IS-normalized matrix effect (IS-ME%) and extraction recovery (ER%) of aztreonam measured at three QC levels.

QC Levels	N° Replicates	Average Matrix Effect (%)	Average IS-Normalized Matrix Effect (%)	Average Extraction Recovery (%)
LQC	30	−34.66	−1.10	98.75
MQC	30	−35.17	−1.25	90.12
HQC	30	−35.05	−1.09	97.81

**Table 9:** Average matrix effect (ME%), IS-normalized matrix effect (IS-ME%) and extraction recovery (ER%) of avibactam measured at three QC levels.

QC Levels	N° Replicates	Average Matrix Effect (%)	Average IS-Normalized Matrix Effect (%)	Average Extraction Recovery (%)
LQC	30	27.98	0.96	96.02
MQC	30	23.85	−0.99	97.51
HQC	30	14.63	−0.79	97.89

**Table 10:** Stability of aztreonam at different storage conditions.

Quality Control		LQC	MQC	HQC
Type of Sample	Tested Conditions	Average Accuracy (Bias %)		
Extracts	Autosampler, day 1	0.0020	0.0003	0.0001
	Autosampler, day 2	−3.13	0.0004	4.55
	Autosampler, day 3	−30.30	−27.00	−25.66
Matrix samples	Freeze–thaw stability 1 cycle	1.60	−1.06	4.54
	Freeze–thaw stability 2 cycle	−15.26	−13.14	−14.62
	Freeze–thaw stability 3 cycle	−17.65	−15.92	−16.31

**Table 11:** Stability of avibactam at different storage conditions.

Quality Control		LQC	MQC	HQC
Type of Sample	Tested Conditions	Average Accuracy (Bias %)		
Extracts	Autosampler, day 1	−5.00	2.40	4.00
	Autosampler, day 5	10.00	4.00	4.80
Matrix samples	Freeze–thaw stability 1 cycle	−1.80	−2.20	−2.70
	Freeze–thaw stability 2 cycle	−3.80	−4.70	−4.20
	Freeze–thaw stability 3 cycle	−8.10	−8.50	−6.20

## Data Availability

The data presented in this study are available upon request from the corresponding author. The data are not publicly available due to privacy restrictions.

## Abbreviations

The following abbreviations are used in this manuscript:

TDM	Therapeutic drug monitoring
UHPLC–qTOF-MS/MS	Ultraperformance Liquid Chromatography-quadrupole-Time-of-Flight-Mass Spectrometry
EMA	European Medicine Agency
MIC	Minimum inhibitory concentration
LC	Liquid chromatography
PK/PD	Pharmacokinetic/pharmacodynamic
DMSO	Dimethyl sulfoxide
QC	Quality control
LQC	Low quality control
MQC	Medium quality control
HQC	High quality control
CI	Continuous infusion
IS	Internal standard
MRM	Multiple Reaction Monitoring
ESI	Electrospray Ionization
DP	Declustering potential
CE	Collision energy
LLOQ	Low Limit of Quantification
ULoQ	Upper Limit of Quantification
S/N	Signal-to-noise ratio
CV	Coefficient of variation
ER	Extraction Recovery
ME	Matrix Effect
AVG	Average
CONC	Concentration
ESBLs	Extended-spectrum β-lactamases
MBLs	Metallo β-lactamases
CAD	Collisionally Activated Dissociation gas
